# The impact of host sex on the outcome of co-infection

**DOI:** 10.1038/s41598-017-00835-z

**Published:** 2017-04-19

**Authors:** Olivia Thompson, Stephen A. Y. Gipson, Matthew D. Hall

**Affiliations:** School of Biological Sciences, Monash University, Melbourne, Victoria 3800 Australia

## Abstract

Males and females vary in many characteristics that typically underlie how well a host is able to fight infection, such as body-size, immune capacity, or energy availability. Although well studied in the context of sexual signalling, there is now growing recognition that these differences can influence aspects of pathogen evolution as well. Here we consider how co-infection between multiple pathogen strains is shaped by male-female differences. In natural populations, infections by more than one pathogen strain or species are believed to be a widespread occurrence. Using the water flea, *Daphnia magna*, we exposed genetically identical males and females to replicated bacterial co-infections. We found that pathogen transmission and virulence were much higher in females. However, males did not simply lower average pathogen fitness, but rather the influence of co-infection was more varied and less defined than in females. We discuss how pathogens may have more fitness benefits to gain, and consequently to lose, when infecting one sex over the other.

## Introduction

Males and females of the same species can be strikingly different from each other. They may differ in many overt traits, such as size, morphology or colouration^1^; but these differences can also extend to the immune response, with one sex often being more severely affected by infections^2,3^. In birds and mammals, for example, males are often more susceptible to infection and develop higher parasite loads than females^4,5^, whereas the reverse pattern is often observed in invertebrates^6–8^. When combined with the diversity of sex ratios, sex biases in dispersal, and general behavioural difference between males and females^1,9,10^, there is ample opportunity for dimorphism between the sexes to influence many aspects of disease epidemiology and evolution^3,8,11^.

Here we consider how differences between males and females can influence a widespread feature of host-pathogen interactions – the influence of co-infections on the outcome of infection. Multiple infections are believed to be more common in nature than infections composed of only a single parasite species or genotype^12,13^. Both theory and empirical studies have shown that the overall virulence of a co-infection can increase, decrease, or remain intermediate to the intrinsic properties of each infecting pathogen^14^. Rarely, however, have these concepts been explored within the context of sexual dimorphism in host resistance and exploitative potential. Co-infection studies typically either explore infection outcomes in only one sex^15,16^, or have not explicitly accounted for males-female differences^17^.

We explored the interplay between host sex and co-infection using the well-studied crustacean *Daphnia magna* and its obligate bacterial pathogen *Pasteuria ramosa* ^15,18,19^. In *Daphnia* up to 50 percent of all individuals in a population can be infected with two or more pathogen species^20^, or eight or more genotypes of the one pathogen species^21^. Male *Daphnia*, however, are significantly smaller, more resistant, and die younger than females^22^; potentially offering a parasite a more difficult pool of resources to exploit than females. To study how the contrasting environments provided by males and females impacted on host and pathogen fitness, we manipulated pathogen competition in genetically identical male and female *Daphnia* (genotype HU-HO-2), using three *P. ramosa* genotypes (C1, C14, and C19) with known infection characteristics. In line with theory^14^, the reduction in host lifespan (relative to uninfected controls) and the production of pathogen transmission spores were used as estimates of co-infection outcomes. We hypothesised that any differences between single and multiple infections seen in females would be dampened in males as a result of the sexual dimorphism in physiological characteristics.

## Results

**Figure 1:**
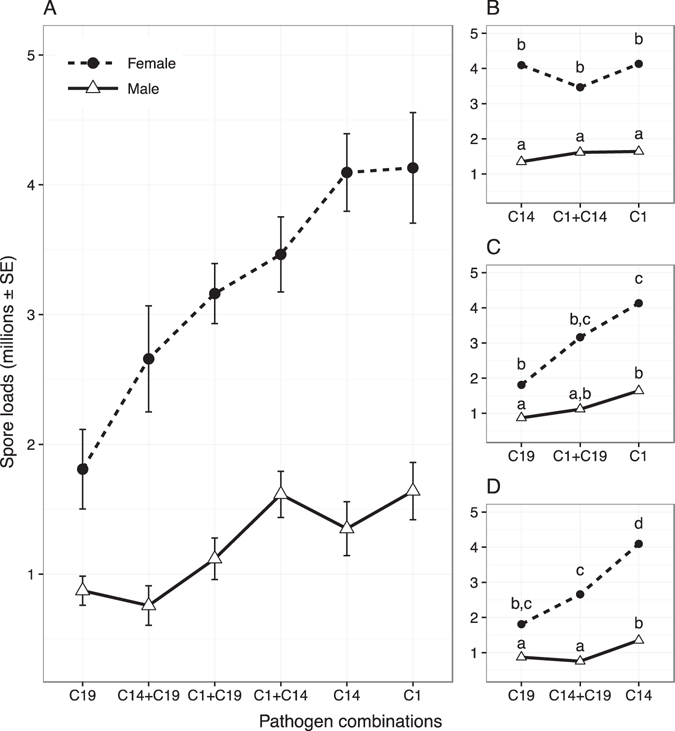
Transmission spores at death for all co-infection treatments. Pathogen combinations in panel A are ordered by spore loads in infected females (left to right, lowest to highest). Panels B to D show the same results subset by the co-infection combinations, with lower-case letters indicating significant groupings via post-hoc t-tests and Benjamini & Hochberg adjusted p-values.

**Figure 2:**
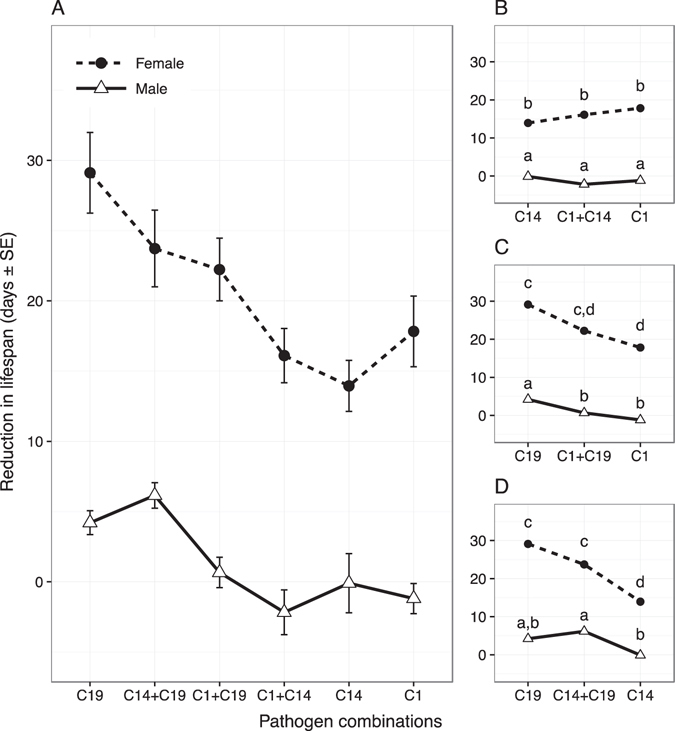
Relative reduction in host lifespan for all co-infection treatments. Pathogen combinations in panel A are ordered by spore loads in infected females (left to right, lowest to highest). Panels B to D show the same results subset by the specific co-infection combinations, with lower-case letters indicating significant groupings via post-hoc t-tests and Benjamini & Hochberg adjusted p-values.

Our results reveal that the production of transmission spores under single and co-infections depended on the sex of the host (Fig. 1). While all pathogen genotypes performed significantly better in females (overall sex effect P < 0.01, Table 1), the influence of the pathogen combinations was sex-specific (overall infection term, P < 0.05, Table 1). This significant interaction was driven by the co-infection combinations where the two pathogens differ in their capacity to produce spores (Fig. 1C and D, see Table 1; but not Fig. 1B). Corresponding to the pattern of pathogen growth, we found that the relative changes in virulence were concordant for each sex (Fig. 2). Overall the average reduction in host lifespan was strongly and negatively correlated with the average production of mature transmission spores (correlation [95% CI]: females −0.93 [−0.99, −0.45]; males −0.98 [−0.99, −0.78]). Although females suffered a much higher reduction in lifespan than males (overall sex effect: P < 0.01, Table 1), there was no evidence for differences between the sexes in how well different pathogen combinations induced virulence (overall interaction term, P > 0.05, Table 1).

**Table 1:** Influence of host sex and co-infection on the production of transmission spores, and the relative reduction in host lifespan. In bold are the overall tests using the entire dataset for the influence of host sex on the outcome of all infection treatments (all single and co-infections together). Below these are the individual analyses for each co-infection combination.

		Infection treatment			Host sex			Interaction		
		F-ratio	df	P-value	F-ratio	df	P-value	F-ratio	df	P-value
**Spore loads at death**		**8.77**	**5, 259**	**<0.001**	**170.41**	**1, 259**	**<0.001**	**3.02**	**5, 259**	**0.011**
*Subset by:*	*C*1 + *C*14	0.33	2, 133	0.723	105.19	1, 133	<0.001	1.632	2, 133	0.199
	*C*1 + *C*19	10.66	2, 125	<0.001	75.30	1, 125	<0.001	4.61	2, 125	0.012
	*C*14 + *C*19	10.01	2, 116	<0.001	67.17	1, 116	<0.001	6.55	2, 116	0.002
**Reduction in lifespan**		**12.18**	**5, 263**	**<0.001**	**288.26**	**1, 263**	**<0.001**	**1.60**	**5, 263**	**0.159**
*Subset by:*	*C*1 + *C*14	0.20	2, 136	0.819	119.69	1, 136	<0.001	0.90	2, 136	0.407
	*C*1 + *C*19	10.96	2, 127	<0.001	182.94	1, 127	<0.001	1.00	2, 127	0.368
	*C*14 + *C*19	11.36	2, 117	<0.001	119.02	1, 117	<0.001	3.44	2, 117	0.036

We compared virulence under coinfection with that expected based on interference competition (intermediate values), competition for resources (higher values than most virulent) or spite (lower values than the least virulent). Post-hoc comparisons, as indicted in Figs 1 and 2, revealed that co-infection scenarios similar to resource competition or spite are least likely, as co-infection never significantly exceeded the most virulent pathogen, nor was lower than the least virulent. Instead, co-infection largely matched the average of the innate characteristics of each infecting genotype in isolation; albeit with some sex-specific differences. When the two pathogens differed in their virulence or spore production, co-infection in females was indistinguishable from the average of the two single infections, and different to that predicted by either the least or most virulent strain (see Table 2). In contrast, coinfection in males was more varied, with one co-infection (C1 + C19) not significantly different to the predicted intermediate values, and another (C14 + C19) more similar to the most virulent and least transmissive strain (C19).

**Table 2:** Tests of equality between the production of spores and the relative reduction in lifespan in the co-infection treatments. Presented are one sample t-tests comparing the co-infection treatment groups against the predicted means of various single infection outcomes. We compared virulence under coinfection with that expected based on interference competition (intermediate values), competition for resources (higher values than most virulent) or spite (lower values than the least virulent). The sign of the t-value indicates whether the co-infection outcome was lower or higher than expected.

		Female co-infections			Male co-infections		
		t-value	df	P-value	t-value	df	P-value
**True mean is equal to the average of the two single infections**							
*Spore loads:*	*C*14 + *C*1	−2.243	29	0.033	0.669	20	0.511
	*C*19 + *C*1	0.830	31	0.413	−0.866	21	0.396
	*C*19 + *C*14	−0.718	26	0.479	−2.329	17	0.032
*Lifespan reduction:*	*C*14 + *C*1	0.113	31	0.912	−0.957	20	0.350
	*C*19 + *C*1	−0.558	31	0.581	−0.781	21	0.444
	*C*19 + *C*14	0.804	26	0.429	4.510	17	<0.001
**True mean is equal to the average of the most virulent strain and least transmissive strain**							
*Spore loads:*	*C*14 + *C*1	−2.182	29	0.037	1.483	20	0.154
	*C*19 + *C*1	5.846	31	<0.001	1.536	21	0.140
	*C*19 + *C*14	2.082	26	0.048	−0.756	17	0.460
*Lifespan reduction:*	*C*14 + *C*1	−0.893	31	0.389	−1.300	20	0.209
	*C*19 + *C*1	−3.086	31	0.004	−3.275	21	0.003
	*C*19 + *C*14	−1.981	26	0.053	2.139	17	0.047
**True mean is equal to the average of the least virulent and most transmissive strain**							
*Spore loads:*	*C*14 + *C*1	−2.305	29	0.029	−0.145	20	0.886
	*C*19 + *C*1	−4.187	31	0.002	−3.268	21	0.004
	*C*19 + *C*14	−3.517	26	0.002	−3.902	17	0.001
*Lifespan reduction:*	*C*14 + *C*1	1.112	31	0.273	−0.615	20	0.546
	*C*19 + *C*1	1.970	31	0.058	1.713	21	0.104
	*C*19 + *C*14	3.589	26	0.001	6.877	17	<0.001

## Discussion

Males and females vary in many characteristics that combine to offer pathogens two environments that differ in their exploitative potential^3^. Here, males of *Daphnia magna* are smaller and more resistant to infection than their genetically identical female counterparts^22^, a pattern common to many other arthropods^6,7^. Our results show that these differences change how well a pathogen can exploit a host, with pathogen transmission (mature spore loads) and virulence (reduction in expected lifespan) being much higher in females. However, the presence of the significant interaction term for pathogen transmission indicates that males do not simply lower average pathogen fitness, but rather the transition from the worst performing pathogen, to the mixed co-infection, to the best performing pathogen was different in each sex.

Emerging from our results is that the best performing pathogens have the most to gain from single infections in females, but also the most to lose from co-infections in the same sex. High-growth pathogen strains (C1 or C14) outperformed their lesser performing competitor (C19) in single infections by 2.1 million spores on average in females, versus only 0.55 million spores in males. As the evolutionary advantage of a pathogen depends its success relative to other competitors, not its absolute transmission, this represents a nearly four-fold improvement in relative fitness. Conversely, the same pathogens also experienced the greatest reduction in transmission success when co-infections occurred in females. Total spore loads for a co-infection were always less than that of the high-growth pathogen strain in isolation; a substantial loss of potential fitness even if the other pathogen was outcompeted completely. We also cannot rule out that the lesser performing pathogen in isolation (C19) is a superior competitor in a co-infection, a fact that would further decrease the fitness of the pathogen ranked best in isolation. Overall, this loss of fitness for high growth pathogens (and potential gain for low growth pathogens) was three-fold higher in females (1.2 million spores) than males (0.41 million spores).

The distinction between males and females was more subtle when considering the virulence of co-infections. For females, virulence in co-infections closely matched those expected by interference competition or immune-mediated competition between competing strains^14^. Overall virulence of a co-infection was firmly intermediate to that of the two competing pathogens, ruling out alternate explanations that predict either an increase (competition for a finite pool of resources, or facilitation via immune depletion) or a decrease (generation of spite) in virulence^14^. Evidence for interference competition-like mechanisms in males was less well-supported, as certain co-infection combinations tracked the overall characteristics of the least virulent pathogen. Given the lack of an overall significant interaction term for virulence, however, it appears that co-infection in males does not fundamentally change the driver of overall virulence, but rather that the patterns of co-infection in males may be less defined or extreme than in females.

As with any form of sexual dimorphism, underlying the observed differences in pathogen virulence and transmission may be a range of morphological, physiological, or immune based processes. In this system, males are more resistant (in terms of infection success) and so presumably also better equipped to limit the growth of a pathogen. For male and female *Daphnia*, however, an obvious point of difference is the divergence in body size^22^, whereby males are approximately 25 percent smaller than females. This could also explain the results we observe; irrespective of any immune response. With the larger body size and greater capacity for gigantism (i.e. pathogen induced growth) in females, there is naturally more resources available for a pathogen to exploit (energy or space for replication, for example). Infections in males may just be more constrained by the host, limiting both the potential fitness gains, and even loses, that a pathogen can derive from infection in this sex.

Our findings divert somewhat from previous studies in this system which have typically assumed that virulence tracks the most virulent co-infecting strain^15^, but increasingly it is becoming clear that co-infections depend as much on the specific genotype of the pathogens involved, as well as that of the host, and their interaction^17,23^. We suggest that heterogeneity between the sexes is an additional factor that is likely to influence co-infection outcomes by reducing the intensity of co-infection scenarios when males are infected. Extending this work to include genetic difference in the degree of sexual dimorphism, which we expect given the strong clonal differences observed in *Daphnia*, would further integrate the study of host-pathogen co-evolution with the evolution of male-female differences (see ref.^3^).

## Methods

The host and pathogen genotypes used in this study were chosen as completely compatible and displayed strong genetic interactions for the severity of infectious disease in earlier studies^19,24^. The host genotype (HU-HO-2) originates from Hungary, while the three pathogen genotypes were C1 originating from Moscow, Russia; C19, derived from North Germany; and, C14 from Finland, Tvärminne. Various combinations of these clones, in some form, have been used previously in studies of co-infection^23,25^.

### Production of male and female *Daphnia*

To reliably induce the production of males and females from a single *Daphnia* genotype, we used a modified protocol based on the work of Olmstead and Leblanc^26^. Briefly, we exposed the parental generation to a juvenoid hormone, methyl farnesoate (Echelon Biosciences, product number S-0153), after their first and second clutches, and then collected the third and fourth clutches for use in this experiment. Individuals were placed in 20-mL of the standard *Daphnia* media (ADaM^27^, modified after^28^), but supplemented with methyl farnesoate at a concentration of 300 μg/L. This media was changed either every two days, or when an animal produced a clutch. Importantly, we found that the hormone had no detectable effect on the subsequent fitness of the offspring.

A short pulse of hormone has been used in previous studies to study male-female differences in genetically identical *Daphnia* ^29^. To confirm that the hormone had negligible impact on the life-history of exposed animals, we conducted a series of experimental trials (see Table 3) using female *Daphnia* raised from mothers that were either exposed to the hormone or raised normally in standard *Daphnia* media. We found that there was no significant difference between the animals collected from treated and untreated mothers in the trait means and variances of both general life-history traits (lifespan and clutch production), and the characteristics of infection (infection rates; spore loads, lifespan, and clutch production of infected animals). Using this approach removes the need to stress mothers (low-light, high-density, or starvation) to induce male production; a process which can also influence infection characteristics^19,30^.

**Table 3:** The influence of the hormone treatment on the fitness characteristics of the following offspring generation. Equality of means was assessed using a least-squares linear model in the case of lifespan and spore loads, and a generalised linear model for the number of clutches produced (Poisson distribution, log link function) and infection rates (Binomial distribution, logit link function). Where appropriate, Levene’s test was used to assess homogeneity of variances between the offspring of untreated and treated mothers. All measures were estimated using a single genotype of both *D. manga* (HU-HO-2) and *P. ramosa* (C14), following the same basic infection process outlined in this study.

	Untreated mothers		Treated mothers		Equality of means			Equality of variances		
	Mean	SD	Mean	SD	F or χ^2^	df	P-value	F or χ^2^	df	P-value
**Trial 1: Characteristics of uninfected animals or controls**										
Lifespan (days)	66.86	5.04	67.36	4.71	0.705	1, 268	0.402	0.570	1, 268	0.451
Clutches produced	11.73	1.68	11.49	1.62	0.334	1, 268	0.563	0.201	1, 268	0.654
**Trial 2: Characteristics of infected animals**										
Infection rates	0.89	0.05	0.86	0.054	0.002	1, 69	0.966	—	—	—
Spore loads (millions)	3.97	1.79	3.89	1.94	0.029	1, 61	0.866	0.076	1, 61	0.784
Lifespan (days)	51.88	11.82	50.55	13.38	0.174	1, 61	0.678	0.137	1, 61	0.713
Clutches produced	3.03	1.28	3.10	0.83	0.022	1, 61	0.882	1.362	1, 61	0.248

### Experimental infection trials

Animals were first raised individually in 60-mL jars containing 50 mL of artificial *Daphnia* medium following standard protocols^18,19^, and fed daily with algae (*Chlorella vulgaris*, up to 5 million cells animal^−1^ day^−1^). At four days old, individual males and females were exposed to 20,000 spores from a single *P. ramosa* genotype (C1, C14, and C19), or 20,000 spores containing an equal mix of two genotypes (C1 + C14, C1 + C19, or C14 + C19, 10,000 per genotype). As per standard approaches, this process was repeated the following day when the animals were five days old (40,000 spores in total) to ensure that an internal immune or physiological response, rather than moulting, contributed to differences in infection (see ref.^31^). In total, there were 12 treatment groups (two host sex groups × [3 single infections + 3 multiple infections]), with 36 animals initially assigned to each, plus 20 unexposed controls per sex (472 initial animals).

Survival was monitored daily and animals frozen on the day of death for the assessment of infection status and spore production using a Neubauer haemocytometer. As expected, overall infection rates were lower in males (mean ± SE: 0.58 ± 0.06; range: 0.35 to 0.70) than females (mean ± SE: 0.83 ± 0.04; range: 0.69 to 0.92). Analysis of variance revealed that infection rates indeed varied significantly with host sex (χ^2^ = 33.18, df = 1, P < 0.01) and the co-infection treatments (χ^2^ = 18.91, df = 5, P < 0.01), but were otherwise largely consistent (interaction term: χ^2^ = 6.13, df = 5, P = 0.29). Due to difference in the average lifespan between males and females (males: 33 days ± 1.9; females: 67 days ± 2.0), we subsequently focused on two traits of common currency: the production of transmission spores at host death; and, the reduction in lifespan as compared to the average of the unexposed controls.

### Statistical analysis

All statistical analyses were performed in R (ver. 3.2.4; R Development Core Team, available at www.r-project.org). Initially there were 472 animals exposed to the co-infection treatments, arising from approximately 36 animals each assigned to 12 treatment groups (two host sex groups × [3 single infections + 3 multiple infections]) plus 20 male and 20 female unexposed controls. However, this sample size was reduced by the end of the experiment as: i) not all animals became infected; ii) they died before day 15 post-infection and so could not reliably have their infection status assessed; or iii) were removed from the study due to sampling errors (see Supplementary Information for more details). Before analyses, we checked for the assumptions of linear models (normality, homogeneity of variances). In the case of the spore loads at host death and the reduction in average lifespan, we used a white-adjusted analysis of variance to correct for unequal variances as implemented using the *car* package of R^32^.

Traits were first analysed using a full-factorial analysis of variance (Type III) with the infection treatment (various single or multiple infections), host sex (male or female), and their interaction as fixed effects. For each trait of interest, we began first with an overall test, using the entire dataset (both single and multiple infection together). If a significant effect was detected (host sex, infection treatment, or their interaction) we then explored each subset of pathogen combinations (e.g. C1 versus C14 versus C1 + C14) to see if specific single versus multiple infections combinations were contributing to the overall pattern. In this way, we avoid issues of multiple testing that would arise if we explored every related combination of single and multiple infections in isolation. Finally, one sample t-tests were used to compare the values for co-infection treatment groups against an expected value based on interference competition (intermediate values), competition for resources (higher values than most virulent) or spite (lower values than the least virulent).

### Availability of materials and data

Datasets supporting the conclusions of this article are available via a figshare repository at https://dx.doi.org/10.4225/03/585732493b9ad.

## Electronic supplementary material

Supplementary file 1
