# Erratum: The impact of host sex on the outcome of co-infection

**DOI:** 10.1038/s41598-017-12310-w

**Published:** 2017-09-28

**Authors:** Olivia Thompson, Stephen A. Y. Gipson, Matthew D. Hall

**Affiliations:** School of Biological Sciences, Monash University, Melbourne, Victoria 3800 Australia

*Scientific Reports* **7**:910; doi:10.1038/s41598-017-00835-z; Article published online 19 April 2017

The original version of this Article contained an error in the Introduction section.

“Males and females of the sam matched those expected by interference competition or immune e species can be strikingly different from each other”.

now reads:

“Males and females of the same species can be strikingly different from each other”.

This error has now been corrected in the PDF and HTML versions of the Article.

