# Supplementary material for: The impact of host sex on the outcome of co-infection
Source: Sci Rep. 2017 Apr 19;7:910. doi: 10.1038/s41598-017-00835-z (PMC5430432; doi:10.1038/s41598-017-00835-z)
Supplement: Supplementary file 1 [file 41598_2017_835_MOESM1_ESM.pdf]

Supplementary material:

The impact of host sex on the outcome of co-infection

Olivia Thompson, Stephen A.Y. Gipson, and Matthew D. Hall*

School of Biological Sciences, Monash University, Melbourne, Victoria 3800, Australia

*Corresponding author. Email: matthew.hall@monash.edu

Table S1: The breakdown in sample sizes for the analysis of infection treatment (various single or multiple infections) and host sex (male or female) on pathogen transmission (mature spore loads) and virulence (reduction in host lifespan).

		**Initial *n***	**Infected (*n*)**	**Infection success (%)**	**Virulence (*n*)**	**Transmission (*n*)**
**Males:**	*C1*	34	22	67	20	20
	C14	37	13	46	13	13
	C19	37	11	35	11	10
	C14 + C1	35	23	67	21	21
	C19 + C1	36	24	70	22	22
	C19 + C14	35	21	61	18	18
	Controls	20	NA	NA	19	NA
**Females:**	*C1*	37	27	73	25	24
	C14	36	32	89	31	31
	C19	36	24	69	23	23
	C14 + C1	36	33	92	32	30
	C19 + C1	36	32	92	32	32
	C19 + C14	37	32	86	27	27
	Controls	20	NA	NA	19	NA
